# Estimating COVID‐19 herd immunity in Japan at the beginning of the seventh wave: Transitioning from a pandemic to an endemic

**DOI:** 10.1002/jgf2.573

**Published:** 2022-08-14

**Authors:** Toshikazu Kuniya, Yasuharu Tokuda, Haruyo Nakamura, Takuhiro Moromizato, Kenji Shibuya

**Affiliations:** ^1^ The Tokyo Foundation for Policy Research Tokyo Japan; ^2^ Graduate School of System Informatics Kobe University Kobe Japan; ^3^ Muribushi Okinawa Center for Teaching Hospitals Okinawa Japan; ^4^ Division of Renal and Rheumatology Okinawa Prefectural Nanbu Medical Center Okinawa Japan

## 1 INTRODUCTION

Natural infections and vaccinations are leading to the acquisition of immunity among the general population in Japan; given current trends, it is unlikely that infections will continue to expand over the long term, and there is little need to impose strict behavioral restrictions. The proportion of the population with immunity because of natural infections has increased significantly due to the spread of the Omicron strain, reaching more than twice the number confirmed and well over 20% of the general population. The proportion of those who have at least partial immunity, including from vaccination, is close to 90%, even when the waning of immunity after vaccination is considered. The peak of the current seventh wave of the epidemic is expected in August, and cases should begin declining rapidly thereafter. What is required in effectively controlling the seventh wave is the rapid testing of high‐risk individuals who have developed symptoms and the administration of antiviral drugs to those testing positive, as well as updating the vaccine profile of those who have not yet received third or fourth shots. For highly infectious but less pathogenic variants like the Omicron, a significant supply of rapid antigen testing and further shortening of the current quarantine period are needed to maintain adequate medical and long‐term care and promote socio‐economic activities.

As of July 25, 2022, the number of reported cases of COVID‐19 infection in Japan exceeds 10 million,^1^ and the aggregate number of vaccinations is approaching 300 million.^2^ The number of reported infections is expected to increase further, as the seventh wave of the epidemic caused by the highly infectious Omicron strain BA.5 has begun at the end of June. Estimating the extent to which herd immunity from natural infections and vaccination has been achieved in Japan is essential not only in predicting the end of the spread of infection but also in considering necessary measures going forward. Here, we present such an estimate using currently available statistics and mathematical models (see reference [3] for estimation results as of March 2022).

## 2 MODELS

Similar to the previous approach,^3^ we consider an SEIR model that divides the population into four sub‐populations: Susceptible (S), Exposed I, Infectious (I), and Recovered I. Depending on the number of vaccinations, each sub‐population is further divided into sub‐groups: unvaccinated (S, E, I, R), once (S1, E1, I1, R1), twice (S2, E2, I2, R2), and three times or more (S3, E3, I3, R3). The waning of immunity in accordance with time that has elapsed following the second vaccination, and the booster effect of re‐vaccination is also taken into account. Model specifications are shown in Ref. [4] The infection rate of the model was derived from data in Ref. [1] and vaccine coverage was estimated based on the data presented in Ref. [2].

## 3 RESULTS

**FIGURE 1:**
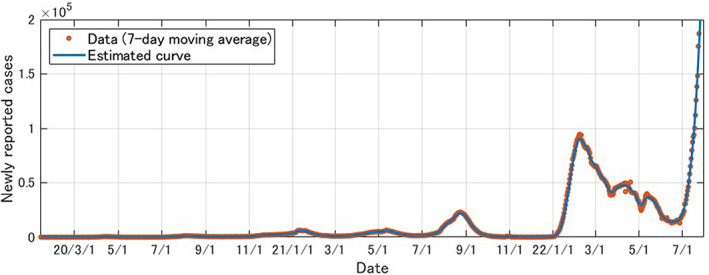
Reported number of daily COVID‐19 infections in Japan (January 14, 2020, to July 25, 2022), published in the Japanese site of the Tokyo Foundation of Policy Research for preview: https://www.tkfd.or.jp/research/detail.php?id=4036

Figure 1 shows the number of reported infections per day (seven‐day moving average) and the epidemic curve of the model.

**FIGURE 2:**
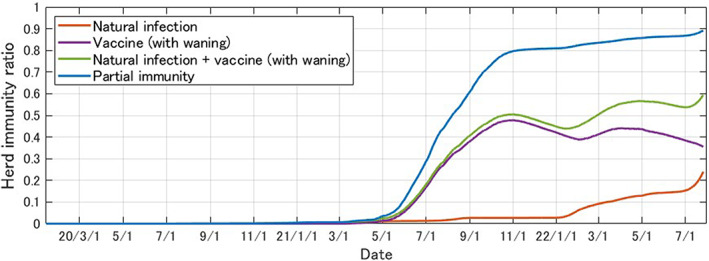
Estimated daily herd immunity rate in Japan (January 14, 2020 to July 25, 2022), published in the Japanese site of the Tokyo Foundation of Policy Research for preview: https://www.tkfd.or.jp/research/detail.php?id=4036

In the previous estimate,^3^ those who had experienced infection were categorized as having “Full” immunity, and those who had been vaccinated were said to have “Partial” immunity. In the present analysis, we regard “Full” immunity as those immunized due to natural infection and call it “Natural” infection. More specifically, this includes the immunized population due to breakthrough infections after vaccination. In addition, we call the population immunized through vaccines alone (and take into account the waning of immunity) as “Vaccine (with waning).” Figure 2 shows the estimated results of the daily herd immunity ratio in Japan under such a classification.

**FIGURE 3:**
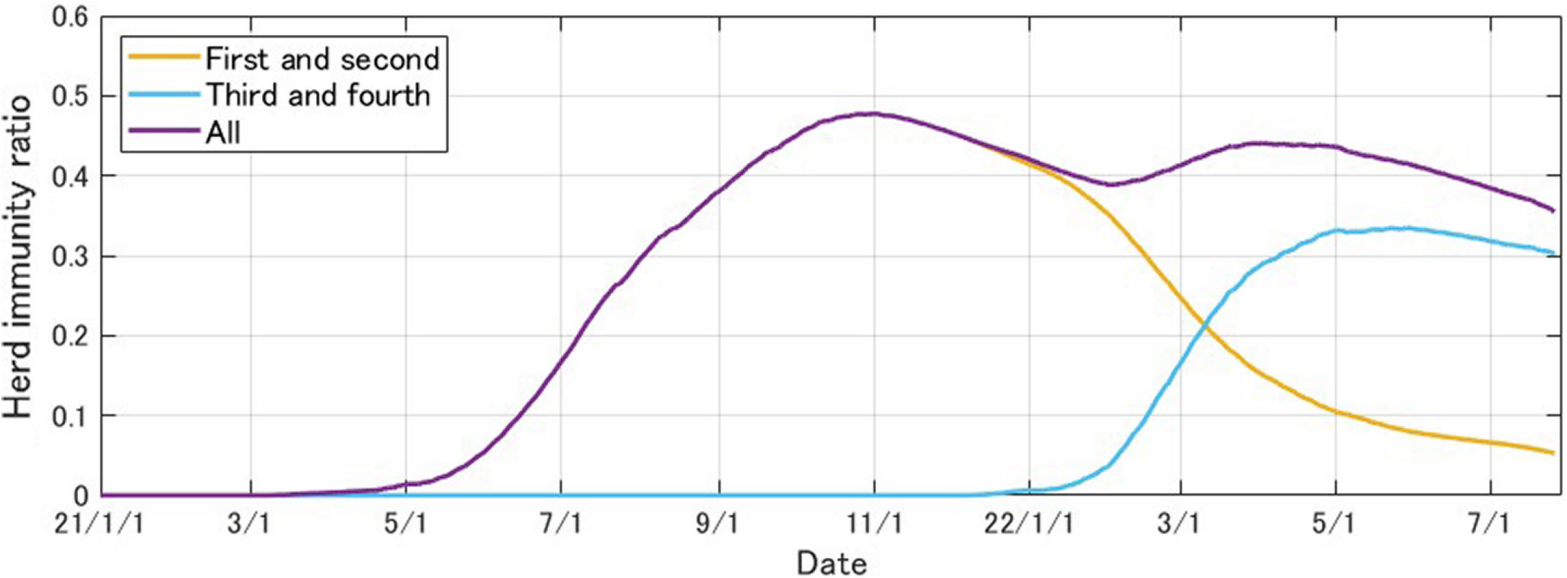
Daily estimates of herd immunity by vaccine status in Japan (January 1, 2021, to July 25, 2022), published in the Japanese site of the Tokyo Foundation of Policy Research for preview: https://www.tkfd.or.jp/research/detail.php?id=4036

Among those in the “Vaccine (with waning)” category, the proportion of those immunized through one or two vaccines is called “first and second,” and the proportion of those immunized with three or four doses is called “third” and fourth.” Figure 3 shows the estimated daily herd immunity ratio through vaccination in Japan since January 2021.

## 4 DISCUSSION

Figure 2 clearly shows that the proportion of the immunized population from natural infection (in red) increased significantly during the sixth and seventh waves of 2022, caused by the highly infectious Omicron strain, and that it is now well over 20%. On the other hand, the proportion of those with partial immunity (in blue), including vaccine‐derived immunity, is close to 90%. Breakthrough infections account for 15% to 30% of naturally infected individuals,^5^ which is only about 3% of those with partial immunity, meaning that the estimated component of immunized proportions is not affected significantly by breakthrough infections. Therefore, it is estimated that the gap between the number of reported infections and the number of hospitalizations, severely ill, and deaths will further expand as people acquire immunity through natural infection and vaccination. In other words, in the current situation, it is not necessary to impose uniform behavioral restrictions, as in the early phase of the COVID‐19 pandemic.

When considering the waning of immunity, however, the proportion of the vaccine‐immunized population (in purple), which was close to 50% at the peak in November 2021, fell below 40% in July 2022. In particular, in December 2021 and June 2022, just before the beginning of the sixth and seventh waves, the proportion of the vaccine‐immunized population clearly decreased. Therefore, it is possible that factors triggering each wave included not just increased infectiousness and the immune escape properties of the variants but also a decline in vaccine‐related herd immunity.

In Figure 3, the proportion of the immunized population from the first and second vaccinations (in yellow) peaked in November 2021, with the proportion of the immunized population receiving third and fourth doses (in light blue) overtaking the former in early March 2022. Currently, immunity among both populations is declining, and the best way to minimize hospitalizations and deaths during the seventh wave is to widely disseminate rapid testing and administer therapeutic drugs to high‐risk individuals who test positive and to promote vaccination among those who have not been vaccinated for the third or fourth time. For highly infectious but less pathogenic variants, such as Omicron, a significant supply of rapid antigen tests and further shortening of the quarantine period are warranted to maintain adequate medical and long‐term care and promote socio‐economic activities.

On the other hand, the overall herd immunity ratio (green in Figure 2), including immunity acquired through natural infection during the seventh wave, is increasing. If the number of hospitalizations or deaths remains relatively small, there is the option of simply waiting for an increase in the herd immunity ratio resulting from natural infections. Considering the acquisition of high immunity and the availability of hospital beds, Japan can be seen as now transitioning from a pandemic to an endemic phase. It is well known that natural infections also increase immunity. There is a need for a scientific consensus on how best to maintain socio‐economic activity by either continuing with regular mass vaccination or through a hybrid approach of vaccination and natural infection.

**FIGURE 4:**
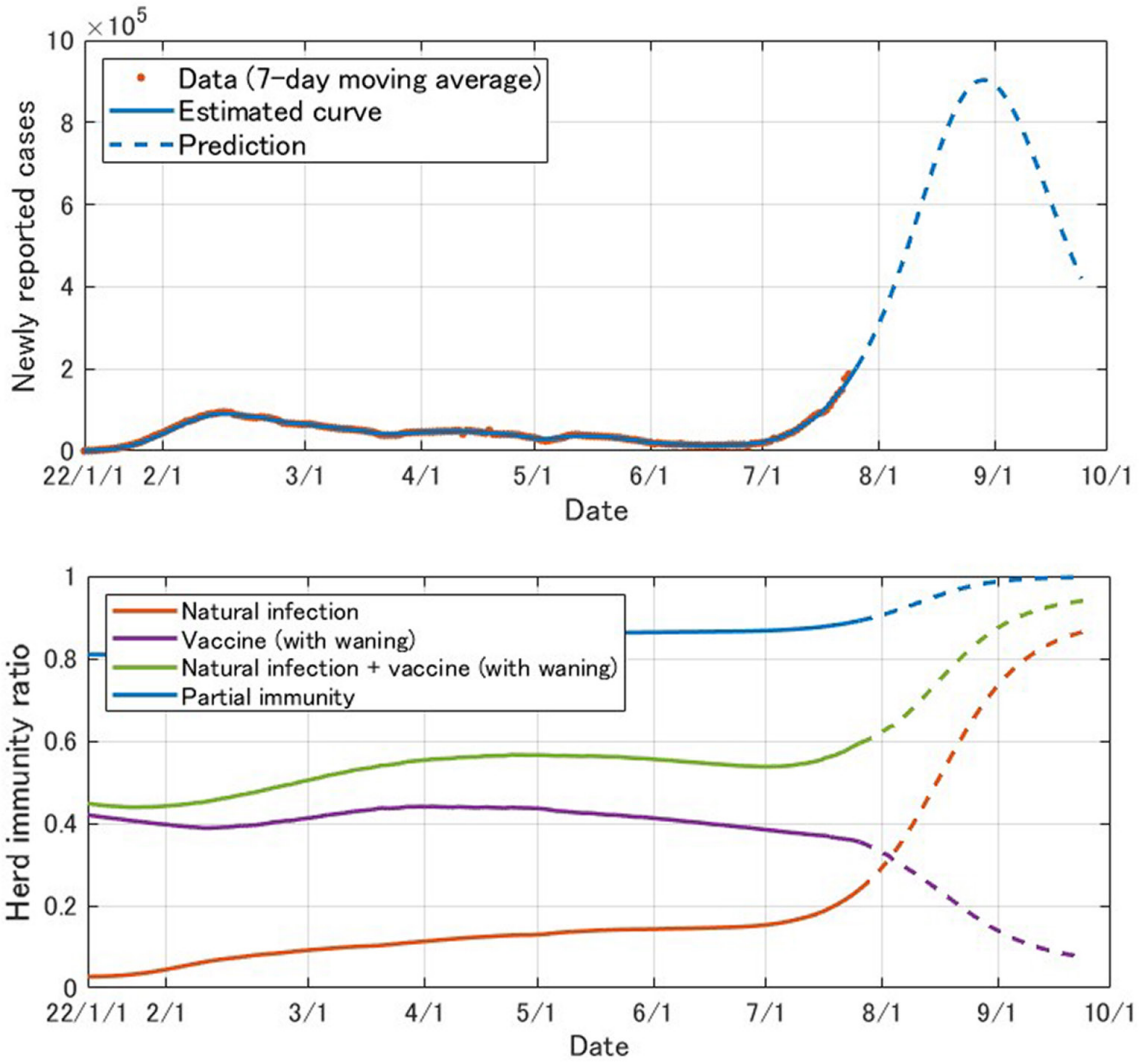
Predictions of daily reported infections and herd immunity ratio in Japan (January 1, 2022, to October 1, 2022; dotted line represents projected figures), published in the Japanese site of the Tokyo Foundation of Policy Research for preview: https://www.tkfd.or.jp/research/detail.php?id=4036

When the basic reproduction number of 2.5^6^ is employed, the critical immunization rate required for ending the pandemic in a typical model^7^ is 60%. Considering only natural infections (red in Figure 2) and vaccination with waning immunity (green in Figure 2), the herd immunity sufficient for the end of the epidemic has not yet been achieved. However, since the proportion of immunity due to natural infection is steadily increasing during the seventh wave, triggered predominantly by BA.5, it is expected that herd immunity will be achieved sooner or later, at least from the simulation model. In particular, given the heterogeneity of infection risk in the general population, it has been pointed out that even lower rates may end the epidemic.^8^ According to the model estimate, the current peak of the seventh wave epidemic will be reached in August, and it will decline rapidly thereafter (Figure 4).

However, if the basic reproduction number is >2.5, the critical immunization rate will also be >60%. In addition, it has been reported that an Omicron infection without vaccination increases the risk of reinfection when compared with breakthrough infections^9^ because only weak immunity is obtained by natural infection. In such cases, the present model, which treats both types of natural infection in the same way, might overestimate the herd immunity acquired from natural infection, making improvement of the vaccination rate more important. Finally, the present model makes various assumptions in the interest of simplification, and the possibility of mutant strains emerging that are more pathogenic than Omicron cannot be ruled out. See reference [3] and its appendix for other possible limitations of the model.

## CONFLICT OF INTEREST

The authors have stated explicitly that there are no conflicts of interest in connection with this article.
